# Mercury induces inflammatory mediator release from human mast cells

**DOI:** 10.1186/1742-2094-7-20

**Published:** 2010-03-11

**Authors:** Duraisamy Kempuraj, Shahrzad Asadi, Bodi Zhang, Akrivi Manola, Jennifer Hogan, Erika Peterson, Theoharis C Theoharides

**Affiliations:** 1 Molecular Immunopharmacology and Drug Discovery Laboratory, Department of Pharmacology and Experimental Therapeutics, Tufts University School of Medicine and Tufts Medical Center, Boston, MA 02111, USA; 2 Department of Obstetrics & Gynecology, Maternal-Fetal Medicine, Tufts University School of Medicine and Tufts Medical Center, Boston, MA 02111, USA; 3 Department of Biochemistry, Tufts University School of Medicine, Boston, MA 02111, USA; 4 Department of Internal Medicine, Tufts University School of Medicine and Tufts Medical Center, Boston, MA 02111, USA; 5 Department of Psychiatry, Tufts University School of Medicine and Tufts Medical Center, Boston, MA 02111, USA

## Abstract

**Background:**

Mercury is known to be neurotoxic, but its effects on the immune system are less well known. Mast cells are involved in allergic reactions, but also in innate and acquired immunity, as well as in inflammation. Many patients with Autism Spectrum Disorders (ASD) have "allergic" symptoms; moreover, the prevalence of ASD in patients with mastocytosis, characterized by numerous hyperactive mast cells in most tissues, is 10-fold higher than the general population suggesting mast cell involvement. We, therefore, investigated the effect of mercuric chloride (HgCl_2_) on human mast cell activation.

**Methods:**

Human leukemic cultured LAD2 mast cells and normal human umbilical cord blood-derived cultured mast cells (hCBMCs) were stimulated by HgCl2 (0.1-10 μM) for either 10 min for beta-hexosaminidase release or 24 hr for measuring vascular endothelial growth factor (VEGF) and IL-6 release by ELISA.

**Results:**

HgCl_2 _induced a 2-fold increase in β-hexosaminidase release, and also significant VEGF release at 0.1 and 1 μM (311 ± 32 pg/10^6 ^cells and 443 ± 143 pg/10^6 ^cells, respectively) from LAD2 mast cells compared to control cells (227 ± 17 pg/10^6 ^cells, n = 5, p < 0.05). Addition of HgCl_2 _(0.1 μM) to the proinflammatory neuropeptide substance P (SP, 0.1 μM) had synergestic action in inducing VEGF from LAD2 mast cells. HgCl_2 _also stimulated significant VEGF release (360 ± 100 pg/10^6 ^cells at 1 μM, n = 5, p < 0.05) from hCBMCs compared to control cells (182 ± 57 pg/10^6 ^cells), and IL-6 release (466 ± 57 pg/10^6 ^cells at 0.1 μM) compared to untreated cells (13 ± 25 pg/10^6 ^cells, n = 5, p < 0.05). Addition of HgCl_2 _(0.1 μM) to SP (5 μM) further increased IL-6 release.

**Conclusions:**

HgCl_2 _stimulates VEGF and IL-6 release from human mast cells. This phenomenon could disrupt the blood-brain-barrier and permit brain inflammation. As a result, the findings of the present study provide a biological mechanism for how low levels of mercury may contribute to ASD pathogenesis.

## Background

Heavy metals such as mercury result in neurological injury that may lead to developmental defects, peripheral neuropathies, and enhanced neurodegenerative changes [1]. Mercurials may be found in various drugs, in bleaching creams, antiseptics, disinfectants, as preservatives in cosmetics, tooth pastes, lens solutions, vaccines, contraceptives and immunotherapy solutions, fungicides, herbicides and in dental fillings, as well as in fish such as tuna due to water pollution [2]. Mercury can cause immune, sensory, neurological, motor, and behavioral dysfunction similar to those associated with Autism Spectrum Disorders (ASD) [2]. The possible role of mercury used as preservative in vaccines [2] has been debated extensively, but most epidemiological studies do not support a causal association between vaccines and autism [3-7]. However, 87% of children included in the US Vaccine Adverse Event Reporting System (VAERS) had ASD [8]. Moreover, a paper based on computerized medical records in the Vaccine Safety Data-link concluded there was "significantly increased rate ratios for ASD with mercury exposure from thiomerosal-containing vaccines" [9]. Mercury has been shown to induce proliferation and cytokine production from T lymphocytes [10]. Mercuric chloride (HgCl_2_) in nontoxic doses induces the release of histamine and cytokines, such as IL-4 and tumor necrosis factor-alpha (TNF-α), from a murine mast cell line and from mouse bone marrow-derived cultured mast cells [11]. HgCl_2 _(100 μM) also enhances immunoglobulin E-mediated mediator release from human basophils [12], and histamine release from a rat basophil cell line (RBL-2H3) [13].

We, therefore, investigated whether HgCl_2 _could stimulate human mast cells, an action that could be enhanced in subjects who already have an atopic background.

## Methods

HgCl_2 _was obtained from Fluka Chemical Corp. (Milwaukee, WI) and was diluted in Dulbecco's phosphate buffered saline (DPBS, GIBCO, Grand Island, NY) on the day of the experiments.

### Human mast cell culture

LAD2 and human umbilical cord blood-derived cultured mast cells (hCBMCs) were cultured as previously described [14,15]. Umbilical cord blood was collected as approved by the Tufts Medical Center's (Boston, MA) Investigation Review Board in tubes containing 10 U/ml heparin, blood was diluted 1:2 with DPBS, GIBCO) containing 2 mM ethylenediaminetetraacetic acid (Sigma, St. Louis, MO). Non-phagocytic mononuclear cells were separated by density-gradient centrifugation using Lymphocyte Separation Medium (LSM) from Organon Teknika Corp. (Durham, NC). The isolation of hematopoietic stem and progenitor cells (CD34^+^) was performed by positive selection of AC133-expressing cells by magnetic-associated cell sorting (MACS) using an AC133 cell isolation kit (Milltenyi Biotec, Auburn, CA) as reported previously [15,16]. CD34^+ ^cells were suspended in AIM-V Medium (GIBCO BRL), supplemented with 100 to 200 ng/ml recombinant human stem cell factor (rhSCF, Amgen, Thousand Oaks, CA), 50 ng/ml IL-6 (Millipore, Temecula, CA) and cultured for 12 to 16 weeks. During this culture period, the cells were washed with DPBS every week and resuspended using fresh culture medium. The purity of hCBMCs was evaluated by immunocytochemical staining for tryptase as previously described [15]. Mast cell viability was determined by Trypan blue (0.3%) exclusion method. LAD2 cells cultured over 10 days and hCBMCs cultured over 12 weeks were used for the experiments.

### Histamine assay

LAD2 or hCBMCs cells were washed with DPBS and mast cell media, once in each. Cell suspensions (5 × 10^4 ^cells per tube, 500 μl/sample) were preincubated with either the neuropeptide substance P (SP, 0.1-2 μM) or anti-IgE (10 μg/ml) as positive controls, or HgCl_2 _(1-10 μM) for 30 min. After the reaction, the cells were centrifuged and the supernatant fluid was collected.

Histamine levels were assayed using EIA histamine kit (# IM2015; Immunotech, Beckman Coulter Company, France) as per the directions. Histamine release was calculated as percent of total.

### β-Hexosaminidase assay

β-Hexosaminidase release, as an index of mast cell degranulation, was assayed using a fluorometric assay as previously reported [17]. Briefly, β-hexosaminidase activity in the supernatant fluid and cell lysates (LAD2 cells, 0.5 × 10^5^/tube, were lysed with 1% Triton X-100 to measure residual cell-associated β-hexosaminidase) were incubated with substrate solution (**p**-nitrophenyl-**N**-acetyl-β-D-glucosaminide from Sigma, St Louis, MO) in 0.1 M citrate buffer (pH 4.5) for 60 min at 37°C. The reaction was terminated by the addition 0.2 M NaOH/0.2 M glycine. Absorbance was read at 405 nm in an enzyme-linked immunosorbent assay reader, and the results are expressed as the percentage of β-hexosaminidase activity released over the total.

### Cytokine assay

LAD2 cells or hCBMCs were washed with DPBS, sterile Tyrode's buffer, and plain culture medium, once in each, and were suspended in complete culture medium without IL-6 (for hCBMCs). The LAD 2 cells or hCBMCs (2 × 10^5 ^cells/well/200 μl) were plated in 96-well, flat-bottom Falcon cell culture plates (Becton Dickinson) and were pre-incubated for 15 min at 37°C in a 5% CO_2 _incubator. The cells were then incubated with either neuropeptide SP (0.1-2 μM) or HgCl_2 _(1-10 μM) for 24 hours at 37°C. Control cells were treated with equal volumes of only the respective culture medium. After the reaction time, plates were centrifuged and the supernatant medium was gently collected from the wells and stored at -80°C until the cytokines were measured by enzyme-linked immunosorbent assay (ELISA) using a commercial kit (Quantikine, R&D Systems, Minneapolis, MN), as reported previously [18]. The minimum detectable levels of VEGF and IL-6 were 5 pg/ml. Cell viability was assessed at 1 hour and at 24 hours using the Trypan blue exclusion method.

### Statistical analysis

All conditions were performed in triplicate, and all experiments were repeated five times (n = 5). Results are presented as mean ± SD. Data from two conditions, such as stimulated and control samples, were compared using the Unpaired 2-tailed Student's t-test. Significance of comparisons is denoted by p < 0.05.

## Results

### Effects of HgCl_2 _on mast cell viability

**Figure 1:**
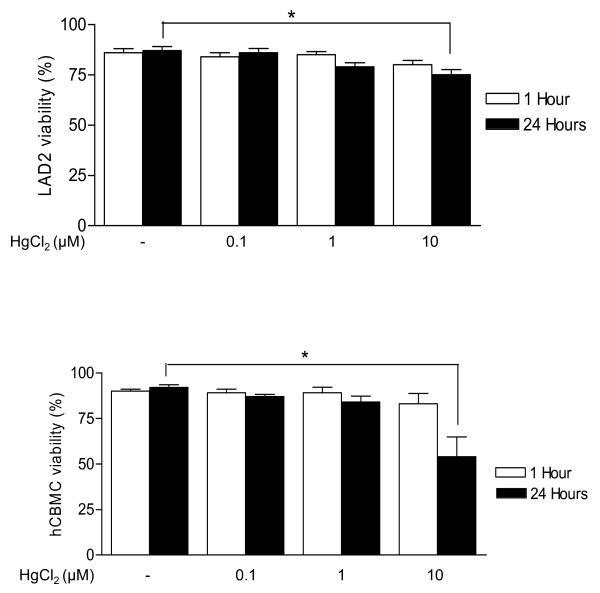
**Effects of HgCl_2 _on (A) LAD2 mast cells and (B) hCBMCs viability assayed by supravital staining with Trypan blue**. LAD2 mast cells and hCBMCs were incubated with HgCl_2 _one hour or 24 hours at 37°C and the viability was assayed (n = 3).

LAD2 mast cells and hCBMCs were incubated with HgCl_2 _for 1 hour or for 24 hours in their respective media, and cell viability was assessed by Trypan blue exclusion. HgCl_2 _reduced viability of LAD2 mast cells in culture medium only slightly (10%), only at concentration of 10 μM, and only after 24 hours of incubation (n = 3, Fig. 1A). HgCl_2 _reduced viability of hCBMCs by 25% at concentration of 10 μM after 24 hours of incubation (n = 5; Fig. 1B).

### Effects of HgCl_2 _on LAD2 mast cell histamine release

**Figure 2:**
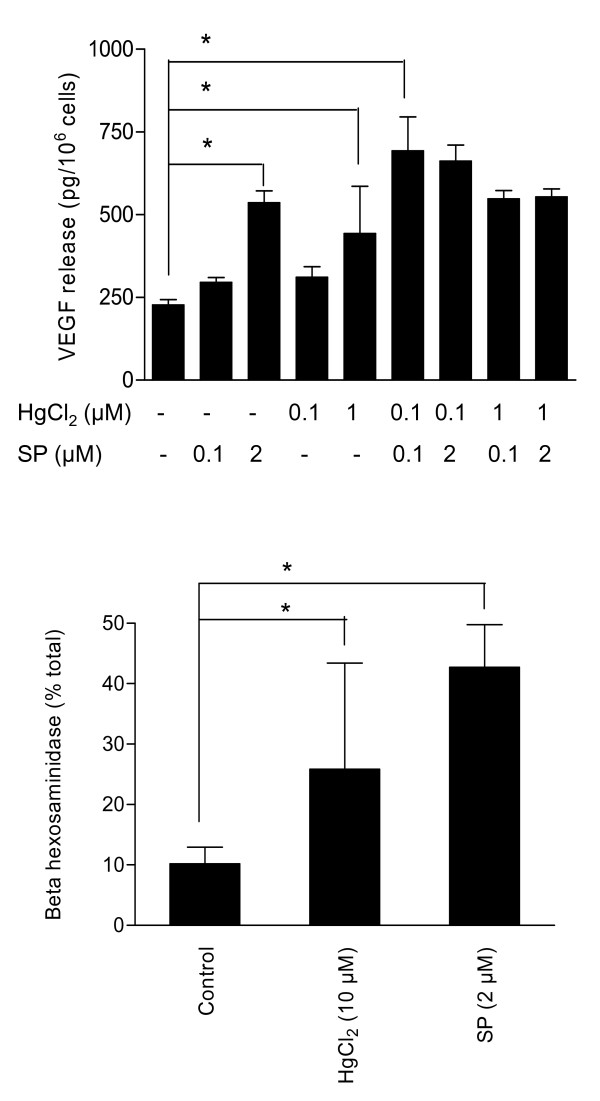
**Effects of HgCl_2 _on (A) β-hexosaminidase and (B) VEGF release from LAD2 mast cells**. LAD2 mast cells were incubated with HgCl_2 _and/or SP as indicated for 10 min for β-hexosaminidase and 24 hours for VEGF release at 37°C. The cells were then centrifuged and the supernatant fluid was collected. VEGF release was assayed by ELISA, while β-hexosaminidase release was assayed spectrophotometrically (n = 5, *p < 0.05).

We first tried to study the effect of HgCl_2 _on mast cell histamine release. We assayed histamine release from LAD2 mast cells and hCBMCs after incubation with HgCl_2 _(1-10 μM) for another 30 min at 37°C in Tyrode's buffer. Addition of HgCl_2 _for 30 min induced statistically significant histamine release from hCBMCs, compared to control cells, at HgCl_2 _concentrations of 0.1 and 1 μM. However, as the results were inconsistent due to interference of HgCl_2 _with the histamine assay, we do not present them. Instead, we investigated the effect of HgCl_2 _on the release of β-hexosaminidase, another secretory granule marker that is released in parallel with histamine. Only 10 μM HgCl_2 _was able to induce a 2-fold increase in β-hexosaminidase release (Fig. 2A, n = 5, p < 0.05).

### Effects of HgCl_2 _on LAD2 mast cell VEGF release

We then investigated whether HgCl_2 _could stimulate release of proinflammatory mediators from mast cells. LAD2 mast cells released significantly more VEGF at HgCl_2 _concentrations of 0.1 and 1 μM (311 ± 32 pg/10^6 ^cells and 443 ± 143 pg/10^6 ^cells, respectively, compared to 227 ± 17 pg/10^6 ^cells for control cells, p < 0.05, Fig. 2B). HgCl_2 _(0.1 μM) had a statistically significant synergistic effect on LAD2 mast cell VEGF release (693 ± 102 pg/106 cells) when added with SP (0.1 μM) (Fig. 2B). Combinations of higher concentrations of HgCl_2 _and SP did not induce any additional VEGF release.

### Effects of HgCl_2 _on hCBMC VEGF and IL-6 release

**Figure 3:**
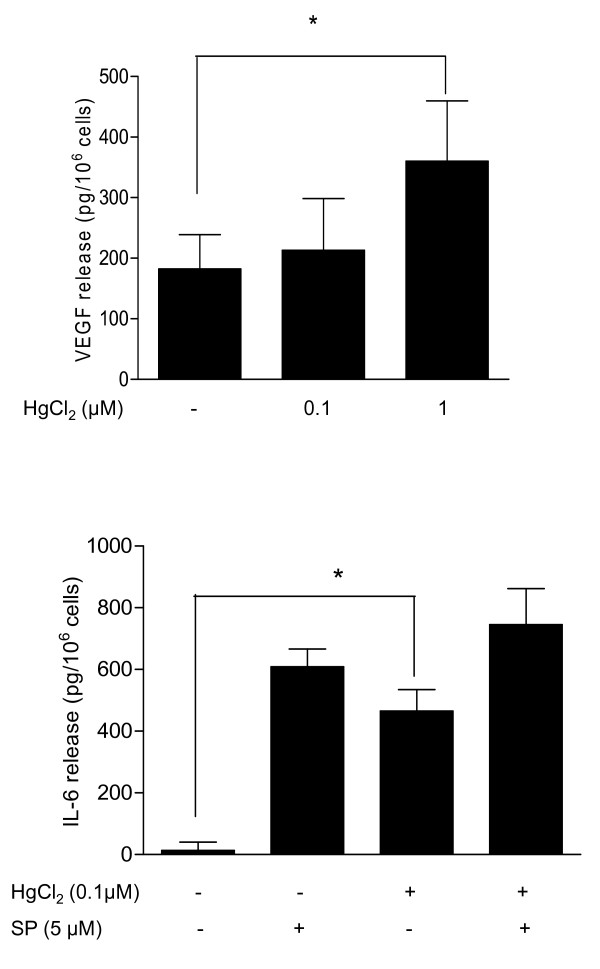
**Effects of HgCl_2 _on VEGF release from hCBMCs**. Mast cells were incubated with HgCl_2 _and/or SP as indicated for 24 hours at 37°C. The cells were then centrifuged and the supernatant fluid was collected. VEGF (A) and IL-6 (B) release from hCBMCs was assayed by ELISA (n = 5, *p < 0.05).

HgCl_2 _(1 μM) also induced release of significantly more VEGF (182 ± 57 pg/10^6 ^cells) from hCBMCs (n = 5, p < 0.05) compared to control cells (360 ± 100 pg/10^6 ^cells) (Fig. 3A).

LAD2 mast cells cannot synthesize IL-6. We, therefore, investigated the effect of HgCl_2 _on IL-6 release from hCBMCs. HgCl_2 _(0.1 and 1 μM) significantly induced IL-6 release (466 ± 57 pg/10^6 ^cells and 204 ± 47 pg/10^6 ^cells, respectively) compared to untreated control cells (13 ± 25 pg/10^6 ^cells, n = 5, Fig. 3B). SP (5 μM), used as a positive control, also significantly increased IL-6 release (609 ± 57 pg/10^6 ^cells) from hCBMCs. Addition of HgCl_2 _(0.1) with SP (5 μM) further increased IL-6 release to 745 ± 117 pg/10^6 ^cells (Fig. 3B).

## Discussion

This is the first report to our knowledge showing that inorganic mercury in concentrations as low as 0.1 μM can induce VEGF and IL-6 release from human cultured mast cells. We also report for the first time that mercury has a significant synergistic effect with SP (0.1 μM) on VEGF release; this amount of VEGF release is higher than what has previously been reported for hCBMCs [19]. One paper has reported that HgCl_2 _can induce release of histamine from primary lung and human leukemic mast cells (HMC-1 cells), but only at toxic levels of 0.33 mM [20]. Here we show that HgCl_2 _induces β-hexosaminidase release, but only at a concentration of 10 μM. Mercury (10 μM) has previously been shown to induce release of β-hexosaminidase, IL-4 and TNF-α from a murine mast cell line and from mouse bone marrow-derived cultured mast cells; the secretion of cytokines mediated by HgCl_2 _is additive to that which follows FcepsilonRI-induced mast cell activation [11]. In contrast, HgCl_2 _does not have an effect on its own on release of histamine and IL-4 from human basophil, but only enhances allergic release at concentrations of 1 and 10 μM [12]. This is also true for IL-4 release from rat mast cells [21]. Clinical symptoms of mercury poisoning may be expected at blood levels of 1 μM [12]. However, brain mast cells may react to lower mercury concentrations, especially in vulnerable patient subpopulations.

Mast cells, by virtue of their location in the skin, respiratory tract, and gastrointestinal system are potential targets for environmental agents with immunotoxic effects [22]. Mast cells are critical not only for allergic reactions, but also important in both innate and acquired immunity [23], as well as in inflammation [24]. In view of the fact that a subgroup of ASD patients have allergy symptoms that do not appear to be triggered by IgE, it is noteworthy that mast cells can be stimulated by non-allergic triggers originating in the gut or the brain [24], especially neuropeptides such as SP [25] and neurotensin (NT) [26]. Once activated, mast cells secrete numerous vasoactive, neurosensitizing and proinflammatory molecules that are relevant to ASD; these include histamine, proteases, VEGF, prostaglandin D_2_, as well as cytokines such as IL-6 [24]. In particular, mast cells can secrete VEGF [27,28], an isoform of which is vasodilatory [29] and is over expressed in delayed hypersensitivity reactions [30]. In fact, mast cells can release VEGF [31], IL-6 [32] and other mediators "selectively" without degranulation [33]. Such mediators could disrupt the gut-blood and blood-brain barriers (BBB) permitting brain inflammation [34]. It is important to note that mercury can cross the BBB through a transport mechanism that can lead to significant brain concentrations, and that can persist for prolonged periods of time [2,35]. Activated brain mast cells can disrupt the BBB [36,37] and further increase brain mercury levels.

The mechanisms of heavy metal neurotoxicity are not fully understood. Mercury increases cytosolic calcium levels in PC12 cells [38], and thimerosal does so in thymus lymphocytes [39]. Mercury may also increase cellular oxidative stress since neurons are highly susceptible to reactive oxygen species (ROS) and neuronal mitochondria are especially vulnerable to oxidative damage [40]. In fact, the primary dietary source of neurotoxic mercury compounds is via the ingestion of methylmercury from fish, which has been previously linked to neurological damage [41].

Mercury's activation of mast cell inflammatory mediator release may enhance allergic reactions in atopic individuals and exacerbate IgE-dependent diseases [12]. Allergic symptomatology is often present in ASD patients [34], and a survey of children with ASD in Italy reported that the strongest association was with a history of allergies [42]. Moreover, a recent study reported increased atopic diseases, as well as elevated serum IgE and eosinophils in Asperger patients [43]. In a National Survey of Children's Health, parents of autistic children reported symptoms of allergies more often than other children, with food allergies showing the greatest difference [44]. A case series study also reported higher rate of food allergies in ASD children [45]. In one study, 30% of autistic children (n = 30) had a history of atopy as compared to 2.5% of age-matched "neurologic controls" (n = 30), but there was no difference in serum IgE or in skin prick tests to 12 common antigens [46], implicating triggers other than IgE. In another study, ASD patients did not have increased incidence of allergic asthma or allergic dermatitis [42], but this study included only ASD patients that were positive to RAST/skin testing. Finally, a preliminary report indicated that the prevalence of ASD may be 10-fold higher [47] than the general population (1/100 children) in mastocytosis patients [48], characterized by increased number of hyperactive mast cells in many tissues, with symptoms that include allergies, food intolerances and "brain fog" [49,50].

Some epidemiological studies have failed to find a significant relationship between mercury exposure from vaccines and autism [3-7]. Nevertheless, 87% of children included in the US Vaccine Adverse Event Reporting System (VAERS) have ASD [8]. Moreover, a paper based on computerized medical records in the Vaccine Safety Datalink concluded there was "significantly increased rate ratios for ASD with mercury exposure from Thimerosal-containing vaccines" [9]. Also, there are a series of epidemiological studies conducted in the USA that have found significant associations between environmental sources of mercury exposure and ASDs [51]. In addition, patients with severe ASD have evidence of significantly increased urinary porphyrins consistent with mercury intoxication [52-55]. Mercury toxicity may also affect critical methylation pathways in vulnerable cells [56].

ASD are a group of pervasive developmental disorders that include autistic disorder, Asperger's disorder, and atypical autism - also known as pervasive developmental disorder-not otherwise specified (PDD-NOS). These are neurodevelopmental disorders diagnosed in early childhood [57]. They are characterized by various degrees of dysfunctional communication and social skills, repetitive and stereotypic behaviors, as well as attention, cognitive, learning and sensory defects [57,58]. ASD cases have increased more than 10-fold during the last decade to a prevalence of 1/100 children [44,57,59]. However, there is no known distinct pathogenesis, there are no biomarkers, and there is no effective treatment [60].

ASD may result from a combination of genetic/biochemical susceptibility and epigenetic exposure to environmental factors, including reduced ability to excrete mercury and/or exposure to mercury at critical developmental periods [2,56]. A number of papers have suggested that ASD may be associated with immune dysfunction [61], while a recent review made the case that ASD may be a neuroimmune disorder involving mast cell activation [34].

## Conclusions

The results of the present study support the biological plausibility of how mercury could contribute to ASD pathogenesis by inducing VEGF and IL-6 release from mast cells, and as a result disrupt the BBB and thus permit brain inflammation. Further studies should investigate the effect of mercury and thimerosal alone or together with allergic and non-immune triggers.

## Abbreviations

ASD: Autism Spectrum Disorders; (DPBS): Dulbecco's phosphate buffered saline; hCBMCs: human umbilical cord blood-derived cultured mast cells; ELISA: enzyme-linked immunosorbent assay; HgCl_2_: mercury chloride; PDD-NOS: pervasive developmental disorder-not otherwise specified; (NT): neurotensin; (SP): Substance P; rhSCF: recombinant human stem cell factor; VAERS: Vaccine Adverse Event Reporting System; VEGF: vascular endothelial growth factor.

## Competing interests

TCT is on the Scientifc Advisory Board of The Mastocytosis Society. TCT is the inventor of US patents No. 6,624,148; 6,689,748; 6,984,667 and EPO 1365777, which cover methods and compositions of mast cell blockers in neuroinflammatory conditions, as well as US patent application No.12/534,571 for diagnosis and treatment of ASD.

## Authors' contributions

This study is based on an original idea of TCT. TCT and DK wrote the manuscript. DK and SA carried out the cytokine, β-hexosaminidase and histamine assays. JH, AM, BZ carried out the viability assays and some mediator assays. EP provided umbilical cord blood. All authors have read and approved the manuscript.
